# *Polygonum multiflorum*-Induced Liver Injury: Clinical Characteristics, Risk Factors, Material Basis, Action Mechanism and Current Challenges

**DOI:** 10.3389/fphar.2019.01467

**Published:** 2019-12-13

**Authors:** Yi Liu, Wenping Wang, Mingyi Sun, Baorui Ma, Linnuo Pang, Yuanyuan Du, Xiaoxv Dong, Xingbin Yin, Jian Ni

**Affiliations:** ^1^School of Chinese Materia Medica, Beijing University of Traditional Chinese Medicine, Beijing, China; ^2^Research Institute of Chinese Medicine, Beijing University of Traditional Chinese Medicine, Beijing, China

**Keywords:** *Polygonum multiflorum* Thunb., liver injury, safety evaluation, clinical characteristics, material basis, action mechanism, current challenges

## Abstract

*Polygonum multiflorum* Thunb. (PM), called *Heshouwu* in China, is a popular Chinese medicine in clinical practice. Several clinical studies have been conducted to evaluate the traditional therapeutic claims and to study the potential therapeutic activity of PM in dyslipidemia and neurodegenerative diseases, highlighting available clinical evidence. In recent years, reports on clinical adverse reactions of Raw Radix *P. multiflorum* (RPM) and *P. multiflorum* Praeparata (PMP) have been on the increase, especially with respect to liver injury. Most liver injury cases had been assessed for causality using RUCAM (Roussel Uclaf Causality Assessment Method) in this paper. However, the components of PM responsible for the reported hepatotoxic effects have not yet been identified. Moreover, many of the reports are contradictory, while studies on the mechanism involved in PM-induced liver damage are not comprehensive. This study was aimed at reviewing the status of research on liver injury due to PM, including clinical characteristics, risk factors, material basis research and mechanism of action, with a view to understanding PM-induced hepatotoxicity, and taking reasonable and effective measures to prevent it. In short, quality control is still one of the major safety problems in TCM drug safety concerns. The model of safety monitoring and risk management of PM drugs is not yet developed. Indeed, the characteristics and risk factors associated with PM require both proper understanding and control of the risk by strengthening standardization of clinical applications, basic science research, quality control in manufacturing, active monitoring methodology and enhancement of international communication and cooperation. Measures should also be encouraged and implemented to promote healthy development of the TCM industry.

## Introduction

Traditional Chinese Medicine (TCM), which plays a significant role in Chinese civilization, is widely used in western societies, Asia, Africa and the Middle East (Huang et al., 2014; Kim et al., 2016; Kasote et al., 2017; Lin et al., 2017; Sumei et al., 2018). TCM relies on natural products, mainly of herbal origin, used either as raw materials for decoction, as prepared herbal medicines, or as formulated traditional medicines. Herbal medicines and other botanicals are often used as complementary and alternative medicines which are believed safe, also has no strict safety and effective standards, should be paid great attention (Moreira et al., 2014; Lee et al., 2015; Wang et al., 2015; Alamgir, 2017; Cho et al., 2017; Cecilia and Orish, 2018; Jing and Teschke, 2018; Teschke et al., 2018). Herb-induced liver injury (HILI) refers to liver damage caused by TCMs, natural medicines and related preparations, resulting in adverse reactions such as dermatitis, nausea, vomiting, diarrhea, thrombocytopenia, coagulopathy, mental state changes, hepatotoxicity, nephrotoxicity, and electrolyte imbalance (Dag et al., 2014; Divya et al., 2016; Ming et al., 2017; Awortwe et al., 2018). However, the degree of HILI varies among different countries and regions (Zhou et al., 2013; Navarro et al., 2014). This variation may be related to the single-center retrospective investigations and different levels of HILI differential diagnosis in each center. Studies have shown that HILI is more common in people over 40 years old, and the prevalence in women is significantly higher than that in men (Takikawa et al., 2009; 2012; Chalasani et al., 2015). Most of the liver damage caused by Chinese herbal medicine is due to the components of herbal medicines. The first Chinese herbal medicine that produced liver damage is *Teucrium chamaedrys* L. Epidemiological investigations have shown 30 cases of patients in France with liver disease caused by *T. chamaedrys*, as a result of which it was removed from the French market in 1992. Since then, several cases have been reported in countries such as Canada and Spain (Larrey, 1992; Haslan et al., 2015). Liver injury caused by other Chinese herbal medicines in recent years has also been reported. These include *Polygonum multiflorum* Thunb. (*He Shouwu* in Chinese, hereinafter referred to as PM) (Lin et al., 2015; Wang et al., 2015; Guo et al., 2016); *Aloe barbadensis* Mill. (Yang et al., 2010; Jeonghun et al., 2014), *Atractylis gummifera* Salzm. ex L.(29), *Centella asiatica* (L.) Urb. (Jahan et al., 2012), *Larrea tridentata* (Sessé & Moc. ex DC.) Coville (Arteaga et al., 2005), *Mitragyna speciosa* (Korth.) Havil. (Harizal et al., 2010), *Morinda citrifolia* L. (Pawlus and Kinghorn, 2007; Yu et al., 2011), *Symphytum officinale* L. (Betz et al., 1994; Rode, 2002), and *Xanthium strumarium* L. (Kamboj and Saluja, 2010; Wang et al., 2011). Thus, the extensive use of these herbal medicines warrants safety measures. Indeed, TCM drug safety monitoring and risk management are becoming increasingly important tasks.

*P. multiflorum* Thunb. (PM), called *Heshouwu* in China, is a popular Chinese medicine in clinical practice. There are two forms of PM decoctions in the *Chinese Pharmacopoeia* (2015): Raw Radix *P. multiflorum* (RPM) and *P. multiflorum* Preparata (PMP*)*. While RPM contributes to detoxification and bowel relaxation, PMP tonifies the liver and kidney, benefits *essence of blood* and *black beard*, and relieves hyperlipidemia, fatty liver, and osteoporosis (Xuan et al., 2007; Wang et al., 2014; Bounda and Feng, 2015). Several clinical studies have been conducted to evaluate the traditional therapeutic claims including anti-inflammatory bioactivity, dyslipidemia, sleep disorders, neurodegenerative disease. For example, PM was significantly tested for the treatment of the hyperlipidemia in a clinical study that enrolled 50 patients. The findings demonstrated that the lipid-lowering effect may be related to its regulating action of the genes involved in cholesterol synthesis and lipoprotein metabolism (Ke et al., 2000). Chen et al. have investigated the therapeutic activity of PM in Alzheimer’s disease (AD) through a clinical trial (Chen L et al., 2010). The findings suggested that the scores for the Ability of Daily Living Scale and the Mini-Mental State Examination were significantly improved in the treatment group compared to the Chinese herb control group and the western medicine control group (P < 0.01). Moreover, in a randomized, Piracetam-controlled, single-center clinical trial, PM was evaluated as monotherapy for vascular dementia (VaD) (Li et al., 2008). The authors found that the total clinical effective rate was 71.25% and that the herbal medicinal had obvious therapeutic effect on VaD, with no relative adverse drug reactions. Therefore, the therapeutic effect of PM on neurodegenerative diseases is quite obvious and widely recognized. In addition, PM is also highly popular in TCM preparations, health foods and toiletries which are applied to *reinforce liver* and *kidney* and *black beard* (Guo et al., 2014; Hu et al., 2014; Lee et al., 2014). According to statistics, there are about 500 Chinese patent medicines and 200 health care products containing PM (State pharmacopoeia committee, 2015a).

In recent years, the incidence of drug-induced liver injury from PM and its related preparations have increased (But et al., 1996; Mazzanti et al., 2004; Jung et al., 2011). Incidents of PM-induced liver damage have led to extensive research interest in China and elsewhere (But et al., 1996; Park et al., 2001; Cho et al., 2009; Furukawa et al., 2010). In the present study, a comprehensive review of the relevant published literature was conducted in order to fully understand the clinical characteristics, pharmacovigilance practice risk and risk factors of PM-associated hepatitis so as to prevent future occurrences.

## Case Reports on Liver Injury Caused by *P. Multiflorum* Thunb.

There is very scanty description of the toxicity of PM in ancient Chinese literature. Only 3 of the 42 ancient books which were published in Early China described the toxicity of PM, and highlighted that the incidence of PM-induced adverse reactions was low. In 1996, Hong

Kong reported the first case of liver injury caused by PM in China (Qi et al., 2013). Then, in 1966, British Medicines and Health Products Administration (MHRA) notified the public of a case of liver damage caused by PM preparation, and clearly stated that PM had safety problems; Canada, Australia, the United Kingdom and other countries made similar sensitizations (But et al., 1996). Multinational drug regulatory authorities have successively introduced policies to regulate and even limit the use of PM and its preparations (Noda et al., 2009; Qi et al., 2013). In 2005, Adverse Drug Reaction Information Bulletin (No.9) of China Food and Drug Administration (CFDA) in a notice on the liver damage caused by *Polygonum* preparation *baishiwan*, recommended that patients should take it under the guidance of doctors, strictly observe symptoms and contraindications, and avoid overdose or long-term usage (CFDA, 2005). Patients were also advised to pay attention to liver function monitoring during the treatment period. The CFDA issued a circular prohibiting people with liver dysfunction from taking five kinds of drugs containing PM: *Yangxueshengfa* capsule, the first *Karasuma*, Radix piece, *shouwu* piece, and *shouwu* particles. These medicines were transferred to prescription drug management with revised specifications in 2013 (CFDA, 2013). In July, 2014, CFDA once again declared that strengthened supervision should be carried out on health foods containing PM, and made the following recommendations: (Kasote et al., 2017) Daily intake of raw products should not exceed 1.5 g, while the daily dose of preparations should not exceed 3.0 g; (Kim et al., 2016) the label of any health food containing PM should specify that the medicine is not suitable for people who have liver insufficiency or who have a family history of liver disease; (Sumei et al., 2018) there should be a precaution to the effect that the PM-containing product is not suitable for long-term excessive usage, and should not be taken along with drugs linked with risk of liver damage; and (Lin et al., 2017) patients must monitor liver function during the medication period (CFDA, 2014a). Moreover, CFDA issued a bulletin to draw attention to the risk of liver injury caused by oral administration of PM, requiring medical staff to fully understand the risk of drug use and patient status before using PM and its prescription preparations, while emphasizing the need to pay attention to the dose and course of treatment, and to avoid simultaneous use of PM and hepatotoxic drugs (CFDA, 2014b). Patients were advised to immediately discontinue the drug and seek medical attention if abnormal liver function was diagnosed. The National Adverse Reaction Monitoring team received more than 10,000 complaints about PM and its preparations, highlighting the issue of liver damage associated with the popularly used herbal medicine in China (National Adverse Monitoring System).

The diagnosis of HILI represents a particular clinical and regulatory challenge with major pitfalls for the causality evaluation. RUCAM (Roussel Uclaf Causality Assessment Method) or its previous synonym CIOMS (Council for International Organizations of Medical Sciences) is a well-established tool in common use to quantitatively assess causality in cases of suspected drug induced liver injury (DILI) and herb induced liver injury (HILI). RUCAM represents a structured, standardized, validated, and hepatotoxicity specific diagnostic approach that attributes scores to individual key items, providing final quantitative gradings of causality for each suspect drug/herb in a case report (Benichou et al., 1993; Danan and Teschke, 2015). An *in situ* analysis of 169 cases of drug-induced liver injury caused by Chinese herbal medicine found that 14 kinds of Chinese herbal medicines exerted potential hepatotoxicity, and the number of cases caused by PM was the highest (Watanabe and Shibuya, 2004; Zhou et al., 2013; Zhu et al., 2015; Jing and Teschke, 2018). Literature on drug-induced liver injury published in Chinese Academic Journals Network Publishing Library revealed the screening of 194 Chinese patent medicines causing drug-induced liver injury, including 100 cases of PM which accounted for 51.6% (Chen and Cai, 1999; Zhi-Feng et al., 2013). In another study, Xiao et al. reported 193,714 cases of liver disease between 2007 and 2016 at The 302^nd^ Hospital, out of which 5,710 cases had abnormal liver function history suspected to be related to drugs. It has been reported that 145 patients took PM or its preparations prior to being diagnosed with HILI (Wang et al., 2015; Wang et al., 2016). The clinical manifestations of liver injury induced by PM and its preparations include jaundice, sclera yellow staining, dark urine, nausea, vomiting, fatigue, weakness, stomach pain, abdominal pain, and loss of appetite (Dong et al., 2014; Xiang et al., 2015). The clinical manifestations of drug-induced hepatitis and viral hepatitis are basically similar. Most patients have a history of repeated medication and repeated onset (Banarova et al., 2012). Studies have revealed that that PM associated with liver damage can occur with no gender orientation, and at any age group (Xiang et al., 2015). In most cases, symptoms of liver damage occur about one month after taking the medicine, and they include fatigue, jaundice, anorexia, and yellow or tawny urine. The liver damage caused by PM in most patients is reversible after discontinuation of PM products, and active treatment can restore liver function, although some patients develop liver failure and die. It is hereby suggested that patients take PM products under the guidance of a physician or pharmacist, and avoid long-term usage or intake of high doses of the drugs. Basic characteristics of RUCAM have been provided in various publications (Danan and Teschke, 2018). The HILI signature should be determined according to the ratio R. In practice, two types of liver injury are considered for evaluation: hepatocellular injury (R > 5) and cholestatic/mixed liver injury (R = < 5) (Teschke, 2018). Furthermore, the HILI caused by PM between 2010 to 2019 referencing all publications that used RUCAM for causality assessment or positive reexposure tests are shown in **Table 1**.

**Table 1:** Patient details recorded from published case reports and case series on liver injury caused by PM.

Reference	Reasons for medication	N. of case (M/F)	Age (Year)	HSW type	Assessment methods	Type of liver injury (number)	Outcome
Liu et al. (2013)	Grey hair	1(0/1)	20	PM	RUCAM	M(1)	Recover
Miao et al. (2013)	Health care, Hyperlipidemia	2(0/2)	52;61	RPM; PMP	NA	C(2)	Recover
Liu et al. (2013)	Vitiligo	1(1/0)	37	PMP	NA	NA	Recover
Gao et al. (2013)	Vitiligo	1(0/1)	14	PMP	NA	C(1)	Recover
Banarova et al. (2012)	NA	1(0/1)	33	Shou Wuwan	NA	NA	Recover
Shao and Li (2012)	Grey hair; Health care	2(1/1)	50;40	RPM; PMP	NA	H(2)	Recover
Hu et al. (2012)	Chronic nephritis	1(1/0)	36	PMP	NA	H(1)	Recover
Zhen and Zeng (2012)	Grey hair	1(0/1)	52	PMP	RUCAM	C(1)	Recover
(Li and Zhang (2012)	Grey hair; Hair loss	2(0/2)	50; 28	RPM	NA	NA	Recover
Chen S. X. et al. (2012)	Grey hair	1(1/0)	57	RPM	NA	C(1)	Recover
Liu et al. (2012)	Grey hair	1(0/1)	56	Yishen Wufa	NA	H(1)	Recover
Sun (2011)	Allergic rhinitis; Health care	2(0/2)	39; 72	RPM	NA	H(2)	Recover
Cao et al. (2011)	Grey hair	1(1/0)	28	PMP	NA	NA	Recover
Liu et al. (2011)	Hair loss	1(1/0)	26	Yangxue shengfa	NA	NA	Recover
Wu and Niu (2011)	Hair loss	1(0/1)	47	Yishen wufa	NA	NA	Recover
Liu et al. (2010)	Hair loss	1(0/1)	50	Shou Wuwan	Chinese Hepatitis Study	H(1)	Recover
Chen M. X. et al. (2010)	Health care	2(1/1)	46; 57	PM tea	NA	H(2)	Recover
Yun et al. (2010)	Grey hair	1(0/1)	51	Shou Wuwan	NA	NA	Recover
Furukawa et al. (2010)	NA	1(0/1)	53	Shou Wupian	NA	C(1)	Recover
Lei et al. (2015)	Hair loss	1(1/0)	26	Jingwu capsule	NA	H(1)	Recover
Xiao (2015)	Hair loss	1(1/0)	34	PM	NA	H(1)	Recover
Zhao (2013)	Grey hair	1(1/0)	39	Jingwu capsule	RUCAM	H(1)	Recover
Yan (2011)	Hair loss	1(1/0)	28	HSW tea	RUCAM	H(1)	Recover
Shen et al. (2010)	Leucoderma	1(0/1)	NA	PM preparation	RUCAM	H(1)	Recover
Li et al. (2015)	Hair loss	1(1/0)	26	Qibao Meiran Wan	NA	NA	Recover
Liu et al. (2015)	Hair loss	2(0/2)	47; 25	PM	NA	NA	Recover
Dong et al. (2014)	NA	18(13/5)	18-63	RPM; PMP	RUCAM	H(18)	Recover
Lian et al. (2013)	Grey hair; Hair loss; et al.	52(30/22)	22-69	PM	RUCAM	H (30); C (9); M (13)	Recover
Zhang et al. (2013)	NA	13(2/11)	35-66	PM	RUCAM	H (6); C (4); M (3)	12 Recoveries; 1 LT
Zhang et al. (2013)	Grey hair; Hair loss;et al.	36(23/13)	24-73	PM preparation	RUCAM	H (21); C (2); M (13)	33 Recoveries; 1 Cirrhosis; 2 Deaths
Ding (2012)	Grey hair; Hair loss;et al.	65(20/45)	34-71	PM preparation	RUCAM	NA	64 Recoveries; 1 Death
Guo (2012)	NA	15(7/8)	18-57	PM	RUCAM	H (8); C (3); M (4)	Recover
Xie et al. (2012)	NA	10(7/3)	36-56	PM	Maria	H (5); C (4); M (1)	9 Recoveries; 1 Death
Song (2011)	NA	26(14/12)	38-71	PM	The seventh edition of diagnostic criteria for internal medicine	NA	Recover
Wang (2011)	NA	20(13/7)	34-67	PM	NA	NA	Recover
Jung et al. (2011)	NA	25(18/7)	24-65	PM	RUCAM	H (18); M (7)	23 Recoveries; 1 LT; 1 Death
Chen S. P. et al. (2012)	Grey hair; Hair loss; et al.	12(7/5)	20-70	PM	RUCAM	H (4); C (4); M (4)	Recover
Liu and Li (2010)	NA	7(2/5)	31-64	PM	NA	NA	Recover
Sheng and Shen (2016)	Health care; Hair loss;Grey hair	50(23/27)	30-70	PM preparation	FDA classification standard	H (30); C (3); M (17)	Recover
Zhu et al. (2015)	Hair loss; Grey hair; Health care	66(31/35)	8-63	PM preparation	RUCAM	H (61); C (1); M (4)	57 Recoveries; 4 Hepatic failure; 4 Cirrhosis;1 Deaths
Guo et al. (2015)	Vitiligo; Hair loss; Grey hair; et al.	15(6/9)	35-65	PM	NA	NA	Recover
Dong (2014)	NA	18(14:4)	19-63	PM	RUCAM)	H(18)	Recover
Wang and Li (2018)	Hair loss;Grey hair; High blood pressure	33(13/20)	17-86	PM preparation	RUCAM	NA	28 Recoveries;1 invalid case; 1 aggravated case; 3 Deaths
Yang (2018)	Hair loss; Grey hair	30(17/13)	33-58	PM	NA	NA	Recover
Chang et al. (2018)	NA	39(16/23)	47.9 ± 14.3	PM preparation	RUCAM	H (36); C (1); M (2)	36 Recoveries;1 Chronic hepatitis case; 1 Cirrhosis
Li (2010)	Grey hair; Hair loss;et al.	11(6/5)	34-58	PM	NA	NA	Recover

## Pathogenic Aspects of Liver Injury Caused by *P. Multiflorum* Thunb.

### Risk Factors of Liver Damage Caused by PM

#### Existence of Inherent Toxic Components

Multiple studies have shown that some chemical constituents of PM may be directly toxic to liver cells, but these constituents have not been fully identified. This will be summarized in detail in part IV.

#### Inappropriate Dosage and Medication Time

The Chinese Pharmacopoeia (2015 edition) stipulates that the dose of RPM is 3–6 g per day, while that of PMP is 3–12 g per day (State pharmacopoeia committee, 2015b). Although the toxicity of PM was rarely mentioned in medical literatures of past dynasties, several reports and information in recent years suggest that excessive and protracted usage of PM can cause toxic reactions in the body (Lin et al., 2015; Yang et al., 2018). In general, the toxicity induced by a medication is proportional to the dose and the duration of exposure to the drug (Bárándi et al., 2010; Yue et al., 2018). People have little understanding of the side effects of PM, and even mistakenly believe that it is safe for a long time (Qi et al., 2013; Yun-Ho et al., 2016). Chen et al. explained the reasons why the doctors of previous generations ignored the toxicity of PM in *Shen Nong’s Herbal Classics* (Chen, 1959; Liu, 1982; Jin et al., 2013). The medical practitioners of today regard PM as the top-up of tonics. In the past 20 years, people have long been biased towards the beneficial effects of PM, neglecting its toxicity (Qi et al., 2013). A long-term toxicity study on RPM revealed that continuous administration of RPM at a dose of 20g/kg for 13 weeks caused definite liver damage which was aggravated after 26 weeks, but was reversed after stopping the drug administration (Zhu et al., 2014). In a study on the degree of liver injury caused by different doses of RPM, rats were continuously administered daily doses equivalent to 20, 50, 100, 200 times of corresponding adult dose for 3 months (Hu et al., 2007). The results showed different degrees of liver damage such as inflammatory cell infiltration, hepatic sinus congestion, and kupffer cell proliferation (Hu et al., 2007; Yun et al., 2015). A comparison between the effects of RPM and its preparata on liver injury in rats found that the raw product produced liver damage when it was close to the clinical equivalent dose, while the preparata produced liver damage at a 4-fold dose of the clinical dose. Thus, long-term use of PM preparata also has certain degree of toxic side effects on the liver of rats (Jie et al., 2012; Wang et al., 2014). Continuously administration of different doses of aqueous extract of RPM showed that the activities and mRNA levels of CYP2E1, CYP1A2 were significantly inhibited in rat liver (Ma et al., 2014; Hao et al., 2015; Chun-Lian et al., 2017). A study of “quantity-time-toxic” relationship of hepatotoxicity induced by *Yangxueshengfa*, a PM preparation showed that serum ALT and AST activities peaked 12 h after administration of the drug, and returned to normal after 48 h (Li et al., 2011). It has been reported that *Yangxueshengfa* capsule at the dose of 2.50–12.00 g/kg significantly damaged liver tissue, while ALT, AST and traumatic brain injury (TBI) increased significantly with increase in dose (Xianfeng et al., 2016). Thus, although the occurrence of adverse reactions may be due to individual differences, medication overdose and long exposure to drug all of which are important risk factors for liver damage in PM or its preparations (Wang et al., 2012; He et al., 2016).

#### Immune Damage or Liver Metabolic Enzyme Genetic Polymorphism

Liver injury induced by PM in clinical application has obvious individual differences, indicating that several susceptibility factors exist in PM-induced liver injury. The traditional toxicological valuation model cannot effectively evaluate the specific liver damage at present. For this reason, Xiao’s team constructed a low-dose LPS-induced drug immune-specific liver injury evaluation model for evaluation of the hepatotoxicity caused by RPM. The multi-component effect of RPM and the mechanism of hepatic injury induced by immune system have revealed the scientific nature of immuno-specific PM-induced liver injury (Dan et al., 2016). However, it is still not clear whether other immunologically active components are also involved in the PM-induced immune specificity.

Analysis of the clinical characteristics of patients with liver injury caused by PM and preparations showed that some patients taking normal or less than the normal doses of PM, and even some patients taking the drug only once in a relatively short period of time, developed liver damage. Several patients have a familial adverse reactions to liver organ damage (Liang et al., 2014; China, 2015; Cui et al., 2016; Cheng-Sheng and Sun, 2017). This is probably due to the presence of hereditary hepatic metabolic enzyme deficiency which in turn promotes the accumulation of toxic compounds in the process of biotransformation and metabolism, leading to PM-induced liver damage. The presence of hereditary deficiency of hepatic metabolic enzymes promotes accumulation of several toxic compounds in the process of biotransformation and metabolism, leading to drug-induced liver damage. If the patient has an allergic constitution, this treats the metabolites and components of PM as haptens. After binding to their macromolecular carriers, the haptens form covalently-bound whole antigens which induce the production of antibodies and hypersensitivity. All of these factors can result in serious liver damage. The phase I and phase II metabolic enzyme genes are important in the development and progression of drug-induced liver injury. The genetic susceptibility of PM-related liver injury may be related to individual differences in phase I and phase II metabolic enzymes (Amacher, 2012; Ma et al., 2014; Feng et al., 2014). Genetic polymorphism in hepatic metabolism enzymes, or patient’s hereditary hepatic metabolic enzyme deficiency can cause the accumulation of active constituents of PM during metabolism and biotransformation, leading to drug-induced liver damage (Yun et al., 2015). A study by Huang et al. found that the mRNA expressions of five subtypes of P450 enzymes in liver tissue decreased in a dose-dependent manner after treatment with aqueous extract of RPM, while high-dose treatment significantly inhibited the expressions of CYP1A2 and CYP2E1 mRNA in rat liver (Mei-Xi et al., 2016; Chun-Lian et al., 2017). In addition, other researchers have found that RPM inhibited the expression of CYP450 in rat liver (Zhang et al., 2015). Clinical studies have shown that genetic polymorphism of CYPlA2 differs between normal population and patients with RPM-induced liver injury, through significant differences in the proportion of CYP1A2*1C. This indicates that the activity of CYP1A2 enzyme is lower in patients with RPM-induced liver injury (Ma et al., 2014). Therefore, it is speculated that the mechanism of hepatotoxicity induced by RPM may be related to the genetic polymorphism of CYP450. Some scholars altered the metabolic capability of the liver using drug metabolizing enzyme inhibitors, so as to investigate the effect of trans-stilbene glucoside (the main component of PM) on the susceptibility models of liver injury. The results revealed that inhibition of drug metabolizing enzymes increased liver injury induced by stilbene glycoside, indicating that PM may exert liver damage in patients with different phase II metabolic enzyme gene polymorphisms or low function of these enzymes (Li et al., 2017). Based on the data of the National Center for Adverse Reactions and the clinical database of the drug-induced liver injury in the 302^nd^ Hospital, Xiao et al. found that the incidence of liver damage induced by PM was low. There may be high-risk groups, suggesting that the liver damage of PM may be similar to heterogeneous liver injury. Endotoxin was used as inducer to make an animal model of specific liver injury and RPM was used as an evaluation drug to investigate the liver damage of rats. The results showed that a large dose of 72.5 g/kg caused no significant liver injury in normal rats, while the clinical dose 2 times equivalent (1.08 g/kg) of RPM caused liver injury in an idiosyncratic liver injury model induced by endotoxin, suggesting that there may be a group of people with idiosyncratic sensitivity to hepatotoxicity of RPM (Chun-Yu et al., 2015; Yue et al., 2018). Subsequently, the team also found that the occurrence of immunologically-specific liver injury in RPM was associated with abnormal inhibition of PPAR-γ pathway and overexpression of related inflammatory factors. Pioglitazone (a PPAR-γ agonist) significantly reverses liver damage. Therefore, it is possible to study Chinese medicines for the attenuation of RPM and the mechanism of attenuation (Lan-Zhi et al., 2017).

In summary, genetic polymorphisms of liver metabolic enzymes, metabolic enzyme defects, and immune stress status of different individuals are closely related to liver damage induced by PM.

#### Improper Processing Technology

Processing technology is important in the culture of TCM due to the advantages of improving the efficacy, modifying the property or reducing the side effects of medicines (Zhou and Zhang, 2006; Yang et al., 2016). There are great differences in the records of processing technologies for RPM, with up to twenty different processing methods. Inadequate degree of processing or inadequate processing methods may cause adverse effects on the liver. The toxicity of RPM is reduced by processing. However, *Yangshoushengfa* capsule, *Anshen Buxue* Liquid, *Runzao* Itching Capsule and *Qibao* Hairdressing Pills, all of which contain RPM, have been reported to cause liver damage, suggesting that inadequate processing may cause RPM toxicity. The use of stilbene glycoside and free anthraquinone contents as the only control indicators of processing is irrational (Yun et al., 2015). According to *Materia Medica* records, “processing of RPM is not nine times, do not sleep its poison. Non-black beans, do not kill its potential.” Modern research has shown that RPM drug content profiles of rat blood were significantly different before and after processing, indicating differences in the liver metabolic pathways. Processing can reduce the hepatotoxicity of RPM, as reflected in serum total conjugated bilirubin (CBIL) and total bilirubin (TBIL) profiles (Ma and Wang, 1991; Yun et al., 2015). The constituents of RPM are dynamically changed during processing. After processing, the combined anthraquinone is hydrolyzed to free anthraquinone with diarrhea-reducing effects. The content of stilbene glycoside in RPM was significantly higher than that in PMP, while the contents of monosaccharides, disaccharides, tannins and phospholipids were gradually decreased with extension of processing time (Liu et al., 2008; Rong-Tsun, 2009; Han et al., 2015). A number of studies have shown that the processing of RPM is closely related to the occurrence of adverse reactions. Yang et al. studied the effects of the classic processing method named *nine-steamed and nine-sun*, and a modern processing method called *black bean juice steaming* recorded in Pharmacopoeia, on the chemical constituents of stilbene glycoside, free strontium and combined strontium. The results showed that the contents of these components resulting from the two methods were close, indicating that different processing methods may not affect changes in the components of RPM (Li and Gao, 2015). On the other hand, Pan et al. found that different processing methods caused changes in the contents or structures of the components of RPM, which in turn led to different degrees of liver toxicity (Liu et al., 2011). In summary, the differences in the processing of RPM, and the simplified processing techniques may be important factors for the adverse effects seen in clinical applications, especially hepatotoxicity. As long as the processing technology of RPM is strictly controlled and standardized, the quality stability of products and the clinical efficacy can be guaranteed, thereby minimizing adverse reactions. It is worth noting that, due to the cumbersome, time-consuming, energy-consuming and long production cycle, many new technologies and methods have been introduced to selectively change the traditional processing technology of RPM. The classic method of processing has not been fully passed down. Peeling, processing taboos, steaming time, and processing accessories which affect the toxicity of RPM, have not received enough attention in the improvement of modern processing technology. This is also a key factor that affects the toxicity of RPM.

#### Medicinal Materials Issue

In many reports of adverse reactions, PM was purchased by patients themselves, and even some were derived from the farmer’s market. Thus, it is difficult to ascertain the authenticity of the PM based only on the patient’s oral account. There have been cases where the rhizome of Chinese monkshood was used as PM. The roots of the genus *Pteridophyte* and *Polygonum* are similar to the roots of PM. Consequently, it is difficult to rule out adverse reactions caused by counterfeit drugs and inferior drugs in many reports of liver damage induced by PM. In addition, ancient books have a detailed record of the origin and planting of PM (Yang, 2010). It is necessary to first of all ensure the quality of the medicine itself, and efforts should be made to use PM sold by regular medical units to ensure safety.

#### Improper Modern Preparation Process

##### Modern Extraction Process Can Cause Adverse Reactions

Herbal medicine is traditionally used in the form of water decoction which contains appreciable amounts of proteins, amino acids, polysaccharides and other components. In contrast, modern Chinese medicinal preparations generally use ethanol as a solvent in the extraction process. The chemical compositions of water decoction and ethanol extraction are different (Li et al., 2010; Lu et al., 2013; Wang et al., 2013). Compared with the former, ethanol can extract a lot of lipid-soluble constituents such as fatty acid components. These may appreciably alter the active ingredient spectrum, thereby increasing adverse drug reactions. The preparation process of RPM included in the 2015 edition of the Chinese Pharmacopoeia contains three methods: water decoction, ethanol extraction and direct infiltration after osmosis and pulverization. The types and contents of chemical components extracted by the different preparation processes are also different. Percolation process is used in the extraction of Ginseng *Shouwu* Capsule, *Baoxin* Tablet, and *Tongmai Yangxin* Oral Liquid. Although the percolation process is beneficial for extracting active components which are heat-sensitive, it will also change the chemical composition of the original prescription, resulting in an adverse reaction. Clinically, *Anshen Bunao* Liquid (water extract), Ginseng *Shouwu* Capsule (alcohol extract), and *Tianma Shouwu* Tablet have all been reported in liver injury, indicating that different preparation methods may cause RPM-linked adverse reactions.

Researchers have summarized the reasons for the adverse reactions associated with the modern preparation process. It is believed that the application of modern preparation technology increases the yield of active ingredients and also increases the yield of toxic components, which may lead to adverse reactions (Zhang et al., 2015). In some studies, Lu et al. compared the differences in hepatotoxicities of different PM extraction solvents, and found that 50% ethanol extract was the most toxic (Wang et al., 2015; Deng-Ke et al., 2017). In addition, Liang et al. compared the differences in hepatotoxicities of ethanol extracts and water extracts, and reported that the toxicity of alcohol extracts was much higher than those of water extracts and medicinal materials, suggesting that hepatotoxic substances of PM may be concentrated in the alcohol extract (Liang et al., 2010). In another study, Huang et al. used ultrasonic assisted method to extract anthraquinones from PM, and determined the optimal extraction conditions by orthogonal test, ignoring the fact that anthraquinones may be the toxic components of PM. This was in essence, a new extraction process in place of the traditional water extraction process which may cause adverse reactions in *Polygonum* preparations (Huang et al., 2015). In addition, stilbene glycosides, anthraquinone and polymerization proanthocyanidins were selected as indices of PM formula granules, which may produce adverse reactions in clinical usage of PM. These call for close attention.

##### Modern Pharmaceutical Technologies Can Cause Adverse Reactions

Modern preparation processes such as biosorption, use of macroporous resin columns, and membrane separation effectively improve the utilization of active components, but also inevitably increase the utilization of toxic components. At the same time, macromolecular components such as starch and polysaccharide are usually removed due to their relatively large molecular weights. They have the general effect of moderating the properties of medicines and their detoxification. Although their effects are generally minor, they may lead to increase in adverse reactions. In a study, Wei et al. used macroporous adsorption resin enriched with stilbene glycoside as the core of sustained-release microcapsules in PM (Rao et al., 2009). This is equivalent to increasing the content of the target component in the unit preparation and increasing the clinical dosage of the drug. This may increase the prevalence of adverse reactions in situations where the toxic component of the PM is unclear.

**Figure 1:**
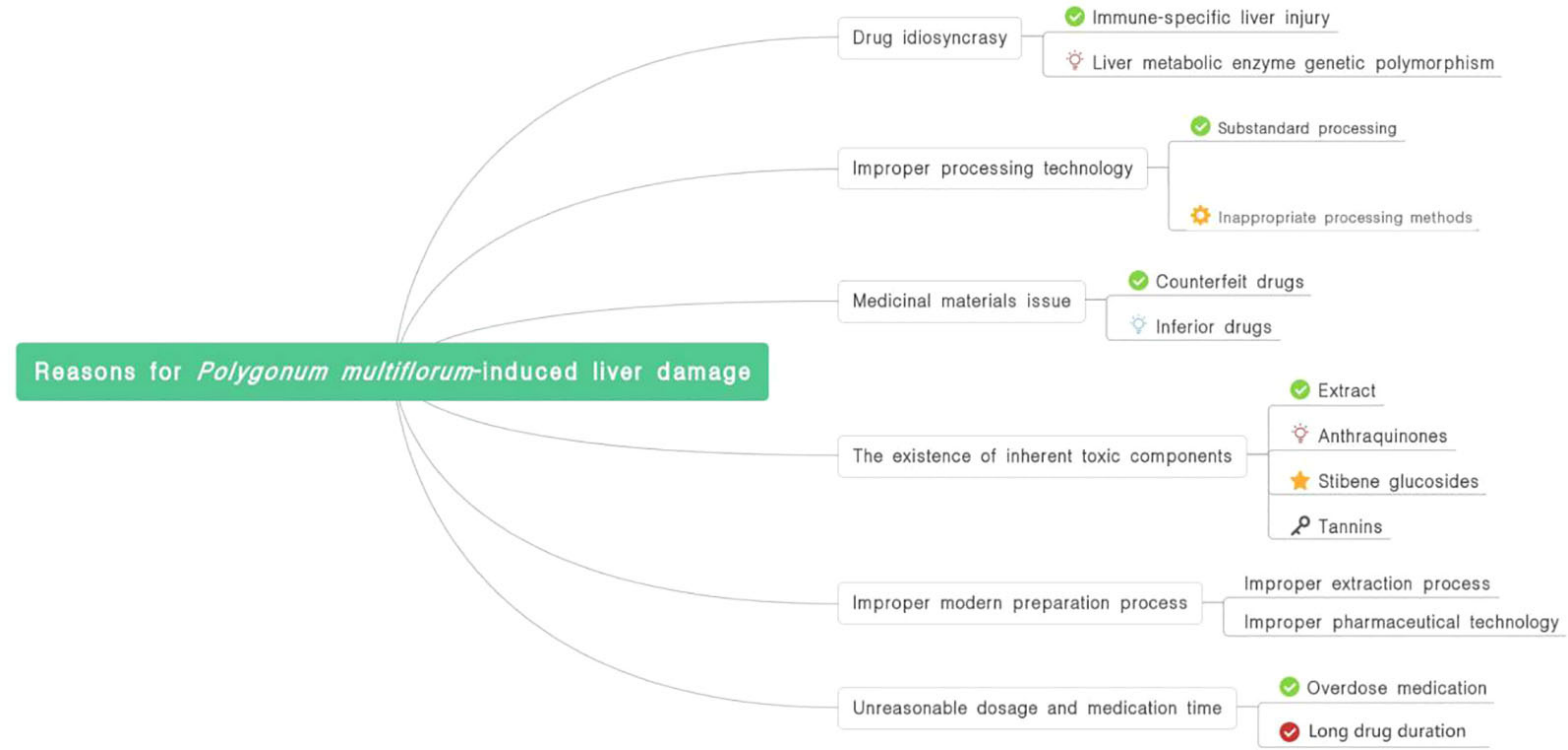
Reasons for *P. multiflorum*-induced liver damage.

The causes of PM-induced liver damage are summarized in **Figure 1**.

### Material Basis Research on Liver Damage Induced by PM

A total of 314 Chinese patent medicines containing PM were retrieved in the Chinese Pharmacopoeia and the Ministry of Standards, including 302 oral preparations and 12 external preparations. At present, among the proprietary Chinese medicines containing PM, only five varieties (*Yangxueshengf*a Capsule, *Shouwu* Pill, *Shouwu* Tablet, *Shouwu Yanshou* Tablet and *Shouwu Yanshou* Granule are managed as prescription drugs, while the rest are over-the-counter drugs (CFDA, 2013). The frequency of use of PMP is much higher than that of raw products among the oral preparations containing RPM. Analysis of adverse reactions have shown that thirteen types proprietary Chinese medicines contained PM, which accounted for 15.66% of varieties of RPM-containing preparations, while 37 types of proprietary Chinese medicines containing PMP accounted for 16.89% of varieties containing processed products (Lin et al., 2015).

The chemical components of PM are mainly stilbene glycosides, terpenoids, phospholipids, tannins and trace elements, but no toxic components have been identified to date. Stilbene glycosides, terpenoids and tannins are the major constituents of PM, and are the primary focus in the search for toxic components. In addition, PM is rich in phospholipids such as lecithin, inositol phospholipids, ethanolamine phospholipids, phosphatidic acid and cardiolipin. However, it is still unclear which component or components are involved in PM-induced liver injury.

#### Extracts

A comparison of acute toxicity in mouse has been made among the whole component, water extract and alcohol extract of RPM with respect to oral lethal dose (LD50), maximum tolerance dose (MTD), maximum lethal dose (MLD), cumulative death number and changes in mouse body weight (Wang et al., 2016). The results showed that the whole component was the most toxic, while the water extract was the least toxic. In a study on the effect of single administration of different components of RPM on “quantity–time–toxicity” in mice, it was found that the activities of ALT and AST peaked at 4 h in mice given higher doses of water extract for the duration of about 24 h (Huang et al., 2011). Serum ALT and AST activities reached peaked at 2 h and continued for 48 h after oral administration of higher doses. After 2 h of administration, the mice showed obvious hepatomegaly and increased liver index. Studies on the toxicity of RPM extract on L-02 human hepatocytes showed that the ethanol extracts of RPM and PMP were significantly more toxic to human hepatocytes than the aqueous extracts, suggesting that ethanol extract of RPM had a higher risk of liver damage (Lin et al., 2015). Ethanol extract of RPM was more hepatotoxic than water extract, due to its higher contents of emodin 8-O-D-glucopyranoside, emodin methyl ether-8-O-D-glucopyranoside, emodin and emodin methyl ether (possible hepatotoxic components) than in water extract (Lv et al., 2015). Some scholars have found that histological sections of mice showed signs of inflammatory response after 3 months of administration of RPM decoction. A study of the hepatotoxicity of different sources of RPM showed that the TCIDso of RPM ranged from 310 to 350 U·g-1, while that of PMP was 130 U ·g-1. Moreover, a comparison of the risk of liver injury caused 8 kinds of Chinese herbal medicines containing PM, using TCIDso as index, showed that *baishiwan* was relatively highly toxic. In a study, bilirubin was used as a substrate for UGT1A1 enzyme to investigate the inhibitory effect of bilirubin glucuronic acid binding on UGT1A1 enzyme activity *in vitro* and *in vivo* during bilirubin metabolism in rat liver. The extract had a strong inhibitory effect on UGT1A1, and the inhibition type was non-competitive inhibition (Qi et al., 2016). In a recent study, the hepatotoxic effects of long-term use of water extracts, alcohol extracts and formula particles of RPM were investigated in rats. The results showed that hepatocytes in different administration groups had different degrees of fatty degeneration and inflammatory cell infiltration. The order of toxicity of RPM was alcohol extract > formula particle group > water extract. It can be concluded that RPM is harmful to the liver, and that the toxicity of RPM is greater than that of PMP, which is consistent with clinical results. Although the hepatotoxicity of processed product is reduced, caution should be exercised in its clinical use.

#### Anthraquinones

The compounds present in PM are mainly emodin, rhein, emodin methyl ether, chrysophanol and aloe-emodin, and their pharmacological effects have been extensively studied. However, excessive use of these anthraquinones stimulate the gastrointestinal tract, resulting in symptoms of diarrhea, abdominal pain, bowel, nausea and vomiting. Severe cases have progressed quickly to paroxysmal tonic convulsions, convulsions, restlessness, and even respiratory paralysis. An analysis of the correlation between PM compounds and cytotoxicity revealed that 10 compounds may be related to the hepatotoxicity of PM, 7 of which are guanidine or glucoside compounds (Lin et al., 2015). Hepatotoxicity could be induced by emodin at high, medium and low doses, indicating that the anthraquinones may be the basis of potential hepatotoxic effects of PM (Can et al., 2015). Studies have demonstrated obvious hepatotoxicities of emodin and rhein. In a study on the correlation between different components of PM and hepatocyte apoptosis, Wei et al. showed that chrysophanol was likely to be the PM component responsible for liver injury. The results showed that emodin and rhein increased damage to L02 and BEL cells in a concentration- or time-dependent manner, while stilbene glycoside had no obvious cytotoxic effect. In addition, emodin, rhein and chrysophanol had no significant effects on the expression of liver CYP450. It has been shown that emodin accumulated in hepatocytes at high-dose repeated administration, with AUC and C_max_ showing upward trends, suggesting causal relationship between emodin and liver-induced damage (Ma et al., 2015). In another study, the liver injury and apoptosis induced by PM were attributed to chrysophanol and other substances. Eight kinds of toxic and controversial monomeric components have been identified in PM using high content analysis (HCA), and it was revealed that anthraquinones were responsible for its potential hepatotoxicity. Moreover, liver damage caused by PM was associated with mitochondrial abnormalities. The UGT1A1 enzyme in bilirubin metabolism has been used to determine the mechanism of hepatotoxicity caused by chemical constituents of PM. It was found that emodin-type anthraquinones selectively inhibited the activity of UGT1A1 enzyme in a structure-activity relationship. In addition, diterpene ketone and anthrone glucosides inhibited the activity of UGT1A1 enzyme. It was speculated that the inhibition may be the mechanism underlying the hepatotoxicity of anthraquinones in PM (Wang et al., 2016). Rhein, emodin and chrysophanol are the main components of anthraquinones in PM, which hepatotoxicity is related to total bilirubin and transaminase contents, inducing liver tissue inflammation, hepatocyte apoptosis and oxidative stress. Yang et al. used zebrafish as a model to study the hepatotoxicity of different extracts and different monomer components of PM (7 anthraquinones, 8 terpenoids, 7 anthrones, 3 cinnamic acid amides and 2 naphthol), and showed that the median lethal dose of emodin was the lowest (Yang et al., 2018). In conclusion, more studies are required to unravel the mechanism of toxicity of PM, and toxicity process in the body.

#### Stilbene Glycosides

At present, relevant research reports on stilbene glycoside-induced liver injury are rare. Liver damage has been reported in SD rats after oral administration of stilbene glycosides (Lv et al., 2011). Moreover, tannins present in PM have been shown to exert liver damage in rats in a synergistic effect with stilbene glycoside. Therefore, various chemical constituents of PM have been shown to be associated with hepatocyte injury. Other researchers used high, medium and low doses of stilbene glycoside for continuous gastric perfusion for 90 days to determine changes in liver enzymes and proteins, and found that the globulins, ALT and AST were increased significantly during the administration period (Zhe et al., 2016). After 15 days of withdrawal, except for the significant decrease of LDH, there were no significant changes in the other indices. This indicates that long-term use of stilbene glycosides may cause some damage to the liver, but liver function can be restored to normal levels after stopping administration of the drug. A comparison of the degree of liver damage by different extracts in a rat model showed that liver toxicity of the ethyl acetate extract was the most severe (Wang et al., 2016; Meng et al., 2017). On further analysis, it was found that cis-stilbene glycoside had the strongest cytotoxicity, suggesting that PM should be stored in the dark during the preservation of alcohol-derived or liquid medicines to avoid or reduce the formation of cis-diphenylethylene and reduce the occurrence of specificity in clinical applications.

#### Tannins

Tannins, which content is up to 15.7%, have been regarded as residue in the extraction process of PM, and have not been taken seriously, thereby ignoring their effect on efficacy and toxicity of PM. As the pharmacological effects of tannins become more prominent, their toxicological studies have begun to receive attention. Studies have found that tannins are important causes of cryptogenic liver damage associated with TCM (Blumenberg et al., 1960; Chandranayagam et al., 2013). Therefore, tannins have also become important aspects of the research on hepatotoxic components of PM. It has been reported that tannins in PM exert toxic effects on the liver. The combination of tannins and stilbene glycoside in different proportions can cause different degrees of liver damage in rats, through a mechanism be related to the decreased secretion of cholinesterase. The combination of tannins and stilbene glycoside damages the liver parenchyma cells through an irreversible synergistic effect. The hepatotoxicity of tannins extracted from RPM has been reported at medium doses, but there were no obvious liver damage at low doses. However, the liver damage was reversible. It has been shown that emodin, rhein, gallic acid, and resveratrol possess biological cytotoxicities, and it was speculated that gallic acid may be the main PM component responsible for liver injury (Yang et al., 2016). A study has found that 70% total ethanol extract of RPM and gallic acid were deleterious to hepatocytes (Chalasani et al., 2014).

### Studies on the Mechanism of PM-Induced Liver Injury

The mechanism of liver injury caused by PM is still unclear. According to current literature reports, liver injury caused by PM comprises intrinsic liver injury and specific liver damage. Rat models were used in most studies, with metabolomics, proteomics and bioinformatics techniques. However, in-depth mechanism of action remains unclear. A study in which serum metabolomics of mice treated with water extract of RPM were analyzed suggested that the liver damage by PM was related to lipid metabolism, amino acid metabolism and excretion of bile acid metabolites (Yun-Xia et al., 2017). Lin et al. demonstrated that the mechanism of liver injury induced by RPM has been attributed to oxidative phosphatization of mitochondrial function and abnormal conduction of TCA cycle signaling pathway, which can lead to hepatocyte apoptosis and abnormal metabolism of bilirubin (Longfei et al., 2017). In addition, extracts of RPM may cause differences in the expression of metabolic enzymes and alter the *in vivo* metabolism of PM components, which may further cause liver damage. A study has investigated liver injury in rats before and after preparation of PM, and screened the sensitive indicators of liver injury. The results showed that the liver injury potential of RPM was significantly lower than that of PMP. Serum DBIL and TBIL which reflect early liver damage can be used as sensitive indicators for liver toxicity monitoring. The mechanism of RPM-induced liver injury has been studied using *kidney yang* deficiency model rats. The study found that RPM administration upregulated TNF-α, while decreasing the activities of Ca^2+^Mg^2+^-ATPase and Na^+^K^+^-ATPase, suggesting that the increase in inflammatory factors caused disorder in mitochondrial function and liver damage. Epigallocatechn gallate (EGCG) is more cytotoxic than catechins and other enamel components, and it can selectively kill cells expressing OATP1B3. Thus, it can lead to an increase in the concentration of substrate for OATP1B3, which can induce hepatotoxicity (Zhang et al., 2013). In addition, mutation of CYP1A2*1C may be related to liver injury caused by RPM, but the specific mechanism involved in its induction of liver damage needs further studies (Benichou et al., 1993). Other possible mechanisms of PM-induced liver injury include long-term intake of PM leading to increased humoral immunity, increased CYP450 content in liver microsomes, suppressed expression of hepatic cytochrome P4502E1, and decreased CYP2E1 activity resulting in inability to clear substances in time. In addition, ethanol extract of RPM caused abnormal inhibition of PPAR-γ pathway and overexpression of related inflammatory factors (Zhou and Zhang, 2006). Liver tissues of rats treated with cis-stilbene glycosides were used to screen differentially expressed genes with gene chip, and it was shown that the TLR4-NFκB pathway may be an important gene pathway for specific liver damage, but the specific mechanism of action requires further research (Wang et al., 2016). In a study aimed at identifying the genetic basis of susceptibility to PM-induced liver injury, it was found that HLA-B*35:01 allele was a potential biomarker for predicting PM-induced damage in humans (Li et al., 2019). This is considered an innovative and progressive discovery

## Reasonable Application of *P. Multiflorum* Thunb.

At present, many classical prescriptions containing PM have been developed as Chinese patent medicine. Different preparation processes have changed the production process and traditional dosage forms, resulting in changes in the composition of the drugs, and also their efficacy and safety. The Chinese Pharmacopoeia represents the level of drug use, pharmaceuticals, and supervision in China. There is need to strengthen people’s understanding of the safety and effectiveness of Chinese herbal medicines containing PM in line with the reality of modern applications. Post-marketing clinical research should be carried out for the 47 varieties of PM preparations; relevant safety information communication and feedback mechanisms should be established, and reasonable use guidelines should be drafted.

Clinically, liver damage due to PM and its preparations has a cumulative effect. In order to reduce the incidence of adverse reactions, it is necessary to take effective measures to ensure the safety of medication. The following suggestions are put forward: (Kasote et al., 2017) Before any patient takes the medicine, they should be fully consulted to understand their family history, allergy history and liver disease history. If the patient has a medical history as above, there is a greater risk of liver damage. Therefore, attention must be paid to rational use of drugs. (Kim et al., 2016) Reasonable control of the duration and dosage of administration should be established. Clinical applications of PM and its preparations should be started from small doses. The dosage should be gradually increased after careful observation of the patient’s medication to prevent various adverse reactions caused by the accumulation of drugs. (Sumei et al., 2018) The monitoring of adverse reactions after drug administration should be strengthened. There is a certain incubation period for hepatotoxicity induced by PM under normal circumstances. Therefore, liver function should be carefully checked after medication. If abnormal liver function is diagnosed, the drug should be discontinued in time. (Lin et al., 2017) Systematic biology research should be fully applied by pharmacists, physicians and researchers to establish a comprehensive database of hepatic damage induced by PM, so as to provide a scientific and reasonable basis for the early diagnosis and prediction of hepatotoxicity. (Huang et al., 2014) Medical personnel should strengthen their understanding of the adverse reactions of TCM, and collect relevant information on the adverse reactions of PM and its preparations with respect to liver injury. Full attention should be paid to the clinical effects of drugs and possible related adverse reactions in order to provide better clinical services. In the current Pharmacopoeia of PM quality standard, the contents of the indicator components (stilbene glycoside, free anthraquinone, combined anthraquinone) which are related to hepatotoxicity indicate only the minimum limits, but are silent on the upper limits. Therefore, it is recommended that the Pharmacopoeia of the People’s Republic of China and the processing regulations set limits on the toxicity indicators in the quality standard of PM. The Chinese medicine decoction production enterprises should strictly follow the regulations in formulating processing technology for PM so ass to ensure its quality.

The safety of TCM drugs is a concern throughout the life period of the drugs. Although pharmacovigilance is absolutely necessary to ensure public safety, measures should also be encouraged and implemented to promote the healthy development of TCM industry. Quality control is still one of the major safety problems in TCM safety concerns. The present study was carried out to review the research status of PM-induced liver injury with respect to clinical characteristics, risk factors, material basis research, and action mechanism. The findings will be useful for further understanding of the hepatotoxicity induced by PM so as to take reasonable and effective measures to prevent it. The model of safety monitoring and risk management of PM drugs is still under investigation. Indeed, the characteristics and risk factors associated with PM require both proper understanding and control by strengthening standardization of clinical applications, basic science research, quality control in manufacturing, active monitoring methodology, and enhancement of international communication and cooperation.

## Author Contributions

YL, JN, and WW contributed to the conception of the study. YL, XY, MS, BM, and YD contributed significantly to analysis and manuscript preparation. YL, XY, XD, and LP performed the data analyses and wrote the manuscript.

## Funding

This research was funded by the National Natural Science Foundation of China (grant number 81673609). The funding sponsors had no role in the design of the study; in the collection, analyses, or interpretation of data; in the writing of the manuscript, or in the decision to publish the results.

## Conflict of Interest

The authors declare that the research was conducted in the absence of any commercial or financial relationships that could be construed as a potential conflict of interest.
